# Hypothesis: Trans‐splicing Generates Evolutionary Novelty in the Photosynthetic Amoeba *Paulinella*

**DOI:** 10.1111/jpy.13247

**Published:** 2022-03-25

**Authors:** Arwa Gabr, Timothy G. Stephens, Debashish Bhattacharya

**Affiliations:** ^1^ Graduate Program in Molecular Bioscience and Program in Microbiology and Molecular Genetics Rutgers University New Brunswick New Jersey 08901 USA; ^2^ Department of Biochemistry and Microbiology Rutgers University New Brunswick New Jersey 08901 USA

**Keywords:** natural selection, *Paulinella*, photosynthetic eukaryotes, protein truncation, Rhizaria, splice leader, trans*‐*splicing

## Abstract

Plastid primary endosymbiosis has occurred twice, once in the Archaeplastida ancestor and once in the *Paulinella* (Rhizaria) lineage. Both events precipitated massive evolutionary changes, including the recruitment and activation of genes that are horizontally acquired (HGT) and the redeployment of existing genes and pathways in novel contexts. Here we address the latter aspect in *Paulinella micropora* KR01 (hereafter, KR01) that has independently evolved spliced leader (SL) trans*‐*splicing (SLTS) of nuclear–derived transcripts. We investigated the role of this process in gene regulation, novel gene origination, and endosymbiont integration. Our analysis shows that 20% of KR01 genes give rise to transcripts with at least one (but in some cases, multiple) sites of SL addition. This process, which often occurs at canonical cis*‐*splicing acceptor sites (internal introns), results in shorter transcripts that may produce 5′‐truncated proteins with novel functions. SL–truncated transcripts fall into four categories that may show: (i) altered protein localization, (ii) altered protein function, structure, or regulation, (iii) loss of valid alternative start codons, preventing translation, or (iv) multiple SL addition sites at the 5′‐terminus. The SL RNA genes required for SLTS are putatively absent in the heterotrophic sister lineage of photosynthetic *Paulinella* species. Moreover, a high proportion of transcripts derived from genes of endosymbiotic gene transfer (EGT) and HGT origin contain SL sequences. We hypothesize that truncation of transcripts by SL addition may facilitate the generation and expression of novel gene variants and that SLTS may have enhanced the activation and fixation of foreign genes in the host genome of the photosynthetic lineages, playing a key role in primary endosymbiont integration.

**Abbreviations**
CDS: coding DNA sequence
crTP: chromatophore transit peptide
EGT: endosymbiotic gene transfer
GO: gene ontology
GSP: gene–specific primers
HGT: horizontal gene transfer
KEGG: kyoto encyclopedia of genes and genomes
ORF: open reading frame
SL: spliced leader
SLTS: splice leader trans‐splicing
UTR: untranslated region

The origin of oxygenic photosynthesis in members of the Archaeplastida (red algae, glaucophytes, green algae plus plants) occurred ca. 1.5 Ga (Yoon et al. 2004, McFadden 2014) via primary endosymbiosis and involved the capture and retention of a beta‐cyanobacterium. These “primary” plastids gave rise, either directly or through subsequent secondary and tertiary endosymbiotic events (Chan and Bhattacharya 2010), to all plastids in photosynthetic eukaryotes, with the sole exception in the rhizarian amoeba *Paulinella*. Of the nine described *Paulinella* species, three (which form a monophyletic clade) harbor an organelle (the chromatophore) derived from an alpha‐cyanobacterial endosymbiont (Marin et al. 2005, Nowack et al. 2008). Early events in the origin of photosynthesis in Archaeplastida are challenging to reconstruct because the genomic evidence is fragmented and provides limited insights into this pivotal event in Earth history (Bhattacharya et al. 2012, Shih and Matzke 2013, Rockwell et al. 2014, Zhang et al. 2017). The *Paulinella* lineage is more amenable in this respect because of the more recent plastid derivation (80–140 Ma; Reyes‐Prieto et al. 2007, Nowack 2014, Delaye et al. 2016, Gabr et al. 2020), making it a model for studying primary endosymbiosis (Yoon et al. 2006, 2009, Nowack et al. 2008, 2011, Nowack and Grossman 2012, Singer et al. 2017, Zhang et al. 2017, Lhee et al. 2019, 2021, Gabr et al. 2020).

The heterotrophic ancestor of photosynthetic *Paulinella* captured and retained a cell from the *Prochlorococcus* *Synechococcus* lineage, establishing the chromatophore (Yoon et al. 2009, Reyes‐Prieto et al. 2010, Rae et al. 2013). The resulting lineages *Paulinella chromatophora, Paulinella micropora*, and *Paulinella longichromatophora* rely on photosynthetic carbon fixation by the chromatophore to provide the energy needed for survival (Kies 1974, Kies and Kremer 1979). Chromatophore acquisition led to high levels of genome divergence among these *Paulinella* species with wide variation in genome size (e.g., ~10 Gbp in *P*. *chromatophora;* Nowack et al. 2016) and 707 Mbp in *P. micropora* KR01 (hereafter, KR01; Lhee et al. 2021). This size fluctuation is explained by increases in repetitive DNA with the KR01 genome assembly comprising 76% repetitive sequences and the *P. micropora* MYN1 assembly, 60.4% repeats (Matsuo et al. 2019). These *Paulinella* species also encode a larger number of exons and longer introns than other Rhizaria and have evolved a novel targeting system to traffic nuclear–encoded proteins to the chromatophore (Singer et al. 2017, Lhee et al. 2021).

Other major innovations include the origin of diurnally rhythmic genes in *Paulinella* *micropora* KR01, of which over 51% have unknown functions (Lhee et al. 2021). These so‐called “dark” genes represent putative novel functional elements that have arisen de novo or through sequence divergence of existing genes to the point where traditional homology detection methods fail to find matches. Given the prevalence of dark, diurnally rhythmic genes and that some of these protein products are chromatophore targeted (Lhee et al. 2021), it appears that *Paulinella* is under selective pressure to evolve novel genes with functions that facilitate the continued integration of the chromatophore into the host metabolism. A mechanism that has not yet been explored and may contribute to the generation of novel sequences in *Paulinella* is spliced–leader trans*‐*splicing (SLTS; Nowack et al. 2016) that involves the transfer of a short (16‐52 bases) conserved spliced leader (SL) sequence derived from specialized non‐coding (nc)RNA to the 5′‐terminus of precursor mRNAs (Zhang et al. 2007, Lasda and Blumenthal 2011, Bitar et al. 2013, Boroni et al. 2018, Matsuo et al. 2018).

Initially reported in trypanosomes (Murphy et al. 1986, Derelle et al. 2010), SLTS has since been described in other euglenozoans (Sutton and Boothroyd 1986, Tessier et al. 1991), metazoans (Vandenberghe et al. 2001, Pettitt et al. 2008, Douris et al. 2010) dinoflagellates (Lidie and van Dolah 2007, Zhang et al. 2007, Bitar et al. 2013), and most recently, Rhizaria (Nowack et al. 2016, Matsuo et al. 2018). The traditional model of SLTS is the addition of an SL sequence occurring at an acceptor site that does not have an associated upstream donor site (known as acceptor‐first syntax; Hastings 2005). These sites are typically located at the 5′‐end of pre‐mRNAs, or between genes on polycistronic transcripts. The addition of the SL sequence at these sites does not interfere with intron removal by *cis‐*splicing at acceptor sites with associated upstream donor sites (donor‐first syntax). Although the spliceosome has a strong preference for SLTS at acceptor‐first sites and *cis‐*splicing at donor‐first sites, in *Schistosoma mansoni*, 43% of SL sequences are added at donor‐first sites (Boroni et al. 2018). Transcripts also occur with multiple SL addition sites, implicating the role of SLTS in generating novel variants (Boroni et al. 2018). Here, we investigate the biological significance of SLTS in KR01 and quantify the frequency of SL addition sites within transcripts to generate hypotheses about the possible role(s) of this process in the evolution of novel functions.

## Materials and Methods

### Cell culture

The *Paulinella micropora* KR01 cell culture used in this study was obtained from Prof. Hwan Su Yoon, Sungkyunkwan University, whose group isolated the cells in 2009 from Mangae Jeosuji (Reservoir), Chungnam Province, South Korea. KR01 cells were grown at 23°C in Fernbach flasks containing 0.75 L DY‐V medium on a 12:12 h light:dark cycle at a light intensity of ~15–20 μmol photons · m^−2^ · s^−1^.

### RNA extraction

Two samples (1 h after lights on and 1 h after lights off) were processed to capture a broad transcript diversity. The cells were swirled in liquid nitrogen for ~30 s before being transferred to ice‐cold 50 mL tubes and centrifuged at 100*g* for 1 min at 4°C. The pellets were resuspended in the supernatant, transferred to ice‐cold 1.5 mL tubes, pelleted by centrifugation at 10,000*g* for 1 min at 4°C, frozen in liquid nitrogen, and stored at −80°C. RNA was extracted using the Qiagen RNeasy Plant Mini Kit according to the manufacturer’s instructions (Qiagen, Hilden, Germany). RNA was eluted with 30 μL RNAase–free water, quantified by absorbance (Nanodrop), and quality controlled using an Agilent Bioanalyzer.

### Cap‐switching RACE and cDNA preparation

To enrich for 5′‐complete transcripts (with or without SLTS), first‐strand cDNA synthesis was done using the SMARTer PCR cDNA Synthesis Kit (Clontech Laboratories, San Jose, CA, USA) according to the manufacturer's instructions. This procedure resulted in single‐strand (ss) cDNA containing the complete 5′‐end of the mRNA with a sequence complementary to the SMARTer primer.

The resulting ss cDNA was amplified by long distance (LD) PCR (Clontech Laboratories), generating ds DNA. Each PCR consisted of a 100 µL reaction volume containing 74 µL of nuclease–free water, 10 µL of 10× Advantage 2 PCR buffer, 2 µL of 50× dNTP (10 mM), 2 µL of 5′ PCR Primer II A, 2 µL of 50× Advantage 2 DNA Polymerase Mix, and 10 µL of the first‐strand reverse transcript product. Initial denaturation was carried out at 95°C for 1 min, followed by 23 cycles of the following thermal‐cycle profile: denaturation at 95°C for 15 s, annealing at 65°C for 30 s, and extension at 68°C for 6 min. About 5 µL of the resulting products were visualized on an agarose gel with ethidium bromide (EtBr).

Preparation of cDNA libraries using TruSeq Nano and Sequencing: libraries of ds cDNA were prepared using the TruSeq Nano DNA Sample Preparation Kit (Illumina) following the manufacturer’s instructions and sequenced on a MiSeq instrument (2 × 150 bp reagents) by Genewiz.

### Searching for SL trans‐spliced transcripts

**Fig. 1:**
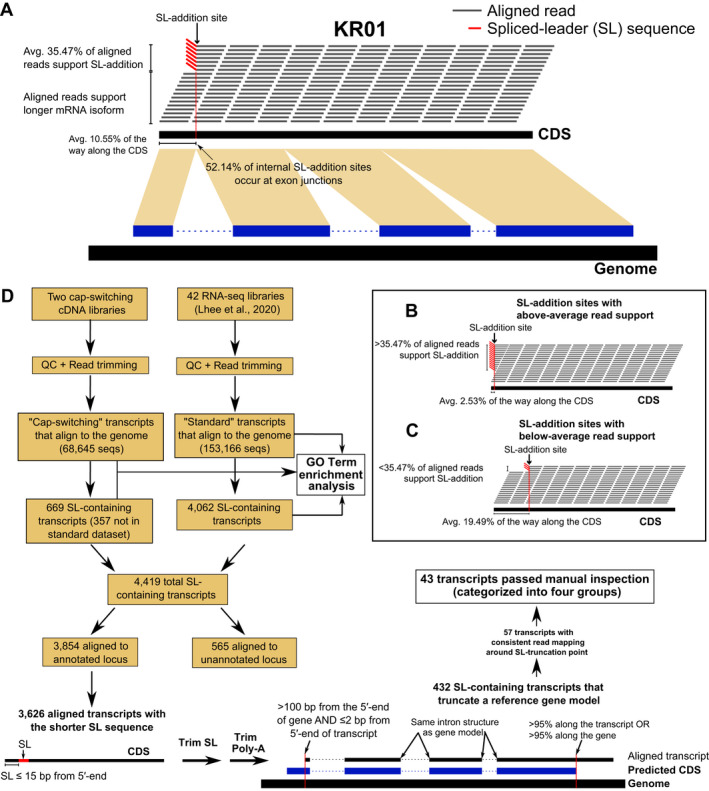
Schematic image showing the approach taken in this study to understand the origin and evolution of SLTS in *Paulinella micropora* KR01. (A) Location and proportion of SL–addition sites in KR01 coding regions. (B) Read‐support for SL–addition sites at the 5′‐end of KR01 CDSs and (C) at more internal sites in the CDSs. (D) Strategy for the cap–switching analysis and outcomes of the work. The blue bars (positioned above the genome) in Panels (A) and (D) represent the CDS of the predicted gene models; the dashed line between the bars represent intron regions. [Color figure can be viewed at wileyonlinelibrary.com]

A schematic of the bioinformatic approach taken to study SL sequences in KR01 is shown in Figure 1. Cap–switching sequence reads were trimmed and assembled into transcripts using CLC Genomics Workbench (Qiagen). The conserved 20‐base *Paulinella* SL sequence (TGGATAATCCGGCTTTTCTG; Nowack et al. 2016, Matsuo et al. 2018) was used to identify transcripts that undergo trans*‐*splicing. SL–containing transcripts were also extracted from an existing transcriptome assembly (hereafter, the standard RNA‐seq dataset; Lhee et al. 2021). To determine the putative functions of these trans*‐*spliced candidates, we utilized BLASTp and the KEGG database to assign them to functional categories.

Transcripts (with and without SL sequences) from the standard and cap–switching assemblies were aligned against the KR01 genome using CLC Genomics Workbench. Transcripts that aligned to the genome were used to predict open reading frames (ORFs) with TransDecoder v5.2.0 (‐m 30; https://github.com/TransDecoder/TransDecoder); ORF prediction was guided by homology information produced by a hmmscan (HMMER v3.1b2) search against the Pfam database (release 33.1; i‐Evalue < 0.001). GO terms were assigned to the predicted proteins using PANNZER2 (sequence filtering set to “query OR subject”; Toronen et al. 2018). SL–containing transcripts aligned to the genome were analyzed for enrichment of GO terms using the topGO R package (Alexa and Rahnenführer 2010), using all aligned transcripts as the background, applying the Fisher’s exact test statistic and the ‘elimination’ algorithm to correct for the hierarchical structure of GO terms.

### Identification of SL–containing transcripts that truncate predicted genes

Because the 5′‐end of transcripts tend to be poorly assembled, only the region (positions 9‐20; CCGGCTTTTCTG) of the SL sequence that was most often recovered by (Nowack et al. 2016) was used in subsequent analysis. Transcripts were retained if they encoded the shorter SL sequence (CCGGCTTTTCTG) with no mismatches, and if the SL sequence started ≤15 bp from the 5′‐end of the transcript (i.e., allowing up to 14 bases to precede the identified SL sequence). If no valid SL sequence was found, the transcript reverse complement was checked (using the same steps). The reverse complement was used for downstream analysis if it encoded a valid SL sequence. The SL sequence and any upstream bases were removed from the transcripts. This resulted in a set of high confidence transcripts that encode the SL sequence at their 5′‐end, are correctly oriented, and lack the SL sequence and any upstream bases.

SL–containing transcripts were trimmed for poly‐A sequences using SeqClean (Chen et al. 2007) and aligned against the KR01 reference genome (Lhee et al. 2021) using PASA v2.3.3 (‐‐transcribed_is_aligned_orient; options used for validate_alignments_in_db.dbi: ‐‐MIN_AVG_PER_ID=95 ‐‐MIN_PERCENT_ALIGNED=90 and subcluster_builder.dbi:‐m = 50; Haas et al. 2003). Only transcripts that PASA classified as having valid alignments against the reference genome (detailed in the alignment.validations.output file) were used for downstream analysis. PASA–validated aligned transcripts were compared against the reference KR01 predicted genes (Lhee et al. 2021) using gffcompare v0.11.5 (Pertea and Pertea 2020), retaining only transcripts with the same intron structure as one of the reference genes (gffcompare class codes “c” and “=”). This produced a set of SL–containing transcripts with intron/exon boundaries that exactly match those of a gene in the reference set; i.e., the SL–containing transcript is derived from the reference gene and not an alternative isoform.

The SL–containing transcripts were aligned against the reference coding sequences (CDS) using BLASTn (v2.9.0; ‐strand plus) and hits between reference genes, and their overlapping SL–containing transcripts (identified by gffcompare) were extracted and analyzed. Hits were retained if they: (i) started ≤2 bp along the SL–containing transcript; (ii) ended >95% along the SL–containing transcript or >95% along the reference gene CDS (this allows for cases where the transcript extends into the 3′ untranslated region of the reference gene); and (iii) started > 100 bp along the reference gene CDS. The point along the CDS where the hit to the SL–containing transcript starts is hereinafter referred to as the "SL–truncation point." This resulted in a set of genes with transcript evidence of an SL sequence being added part way along the reference CDS.

### Assessment of predicted genes to identify mispredicted regions

RNA‐seq (Lhee et al. 2021) reads from KR01 (PRJNA568118; both control and high light samples) were trimmed using Trimmomatic v0.38 (ILLUMINACLIP:adapters.fa:2:30:10 SLIDINGWINDOW:4:5 LEADING:5 TRAILING:5 MINLEN:25; Bolger et al. 2014) and mapped against the reference CDS using bowtie2 v2.3.5.1 (‐‐very‐sensitive‐local; Langmead and Salzberg 2012). For each CDS with an overlapping SL–containing transcript, the region from 100 bp upstream of the SL–truncation point to 50 bp downstream was checked for low or inconsistent read mapping. If the SL–truncation point was <200 bp along the CDS, then the upstream region was set to halfway between the SL–truncation point and the 5′‐ends of the CDS. For each region, starting at the 5′‐end, a sliding window of 20 bases (step size 1 bp) was investigated for reads that mapped from one end of the window to the other (i.e., reads that align over the whole region). If a window had <10 reads that passed the above criteria, it was considered as evidence for the first base at the 5′‐end of the window having been affected by misprediction. Genes with SL–truncated transcripts with regions found to contain mispredicted bases using this approach were discarded. Further manual inspection was performed, removing genes with uneven read coverage or with high levels of mismatches in aligned reads near the SL–truncation point. RNA‐seq reads from *Paulinella* *chromatophora* (NCBI SRA: SRX1624577) were trimmed using Trimmomatic and aligned against the available assembled transcripts (Nowack et al. 2016) using bowtie2 (both programs were run using the same parameters as for KR01).

### Detection of mRNA recycling

To identify putative cases of mRNA recycling (Slamovits and Keeling 2008), both the full length and conserved regions of the *Paulinella* SL sequence were queried (using BLASTn v2.9.0; *e*‐value 1,000, ‐task blastn‐short) against the KR01 reference genome. Only hits with a query coverage of >70% were retained and compared against the locations of predicted reference genes. Genes with SL sequence hits to the genome <100 bp upstream of the start of the gene were considered candidates for mRNA recycling. Candidate genes were manually checked for consistency between the RNA‐seq data (aligned using HISAT2 v2.1.0; Kim et al. 2015) and the reference gene models.

### Alternative start sites, aligned reads encoding the SL sequence, domain annotation, and visualization

**Fig. 2:**
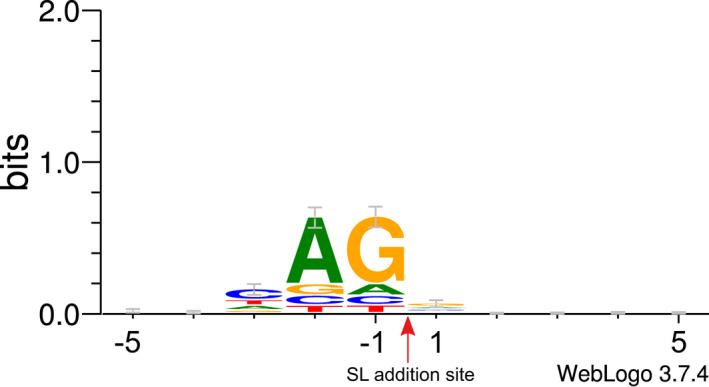
Sequence logo analysis of 1,358 SL addition sites identified in KR01 transcripts that do not overlap with exon splice sites. The CDS sequence 5 bp up‐ and downstream of the SL addition sites is shown as a sequence logo where the SL sequence is added at the position shown with an arrow. [Color figure can be viewed at wileyonlinelibrary.com]

Alternative in‐frame start sites were defined as the positions of methionine within the proteins of genes with SL–truncated transcripts. Additional SL addition sites (in both KR01 and *Paulinella* *chromatophora*) were identified using the bowtie2 locally aligned RNA‐seq reads filtered such that only read pairs where each read was “properly paired” (as determined by the bowtie2 aligner), had ≤5 bp of soft‐clipping at their 3′‐end, and that had an edit distance (optional field code “NM” in SAM output) of ≤5 bp were retained. This extra filtering was performed so only SL addition sites supported by concordantly aligned reads were identified; soft clipping at the 5′‐end of reads was allowed because this could be caused by the presence of the SL sequence in one or both of the reads in a pair. Aligned reads that passed the filtering criteria were checked for the short SL sequence (CCGGCTTTTCTG); if found, the first position along the CDS, after the identified SL sequence was reported. If the SL sequence was encoded in a soft clipped region of the read, then the position along the CDS of the first mapped base was reported. Only positions with >10 reads (Boroni et al. 2018) encoding SL sequences and that were further than 100 bp from a mispredicted region were considered for downstream analysis. Protein sequences of genes with predicted SL–addition sites were annotated using InterProScan v5.44‐79.0. Three sets of features were derived from the InterProScan annotations: (i) functional domains from CDD, PANTHER, Pfam, PRINTS, ProSiteProfiles, SMART, SUPERFAMILY, and TIGRFAM (filtered using *e*‐value <1 × 10^−5^, except for features from ProSiteProfiles which does not report an *e*‐value); (ii) transmembrane domains from Phobius and TMHMM (retaining only annotations with the keywords "TRANSMEMBRANE" or "TMhelix"); and (iii) coil regions from COILS. Within each set, overlapping features were merged using bedtools (Quinlan and Hall 2010). Signal peptides were also predicted in the protein sequences using MITOPROT II (Claros and Vincens 1996), iPSORT (Bannai et al. 2002), and DeepLoc‐1.0 (Almagro Armenteros et al. 2017). SL addition sites downstream of the previously described functional or structural features were retrieved (using “bedtools clostest ‐t first ‐D a ‐id ‐io ‐a SLsites.bed ‐b features.bed”); the region spanning the 5′‐end of the upstream feature to the SL addition site were checked for mispredictions using the approach described above. SL addition sites that overlapped with functional or structural features, and that were positioned >60 bp from there 5′‐end of the overlapping features were retrieved (using “bedtools clostest ‐t first ‐D a ‐a SLsites.bed ‐b features.bed,” retaining only cases where the two returned regions overlap) and checked for mispredictions. SL–truncated transcripts from genes of interest were visualized in R using the Gviz package v1.30.3 (Hahne and Ivanek 2016). The sequence logo of SL addition sites (Fig. 2) was constructed using WebLogo 3 (Schneider and Stephens 1990).

### Verification of SL–containing transcripts

For RT‐PCR, amplification was performed using SL– and gene–specific primers (GSP). The following primers were used: SL (F: TGGATAATCCGGCTTTTCTG), g63591(R; CATTTAGCAGGTGGTTCAGG), g81093 (R: AGAGGTAAACCATCAGCAAGAC), g19409 (F: AAGGGCGAAGAGGACGTA, R: GCCCCACTTGAACCTCTTT), and g851 (F: GTACCAATCAACGCTGTCAAAG, R: CATGGCCGATTGAAAGTTG). Original uncropped gel images are shown in Figures S1 and S2 in the Supporting Information.

### Quantification of SL addition site containing gene expression levels

Expression level quantification was calculated for the control light samples only using RSEM v1.3.3 (‐‐paired‐end ‐‐bowtie2 ‐‐strandedness reverse ‐‐calc‐ci ‐‐calc‐pme ‐‐estimate‐rspd; Li and Dewey 2011) and bowtie2 v2.3.5.1 (Langmead and Salzberg 2012). The transcripts per million (TPM) for each gene was calculated using the expected counts from RSEM and the CDS length. The average TPM for each gene across all control light replicates was compared between genes with and without SL addition sites using a two‐sample t‐test assuming unequal variance.

### Identification of SL sequences in the genome of *Paulinella* species

The genome assemblies of KR01 (Lhee et al. 2021), *Paulinella* *chromatophora* (Nowack et al. 2016), and *P. ovalis* (a heterotrophic lineage; six single‐cell genome assemblies were combined and analyzed together; Bhattacharya et al. 2012) were searched for SL sequences using BLASTn (v2.10.1; ‐evalue 1000 ‐task blastn‐short). Hits were considered to cover the full SL sequence if they started at the first (for KR01 and *P. ovalis*) or second (for *P. chromatophora*) position and finished at position 20 along the SL; the difference in the start position of the different species is because the *P. chromatophora* SL has a cytosine at its first position rather than a thymine (Nowack et al. 2016). Hits were considered to partially cover the SL sequence if they ended at position 20 along the SL but did not start at the first or second positions. The 3′‐end of the SL is the most conserved, which is why the partial hits had to end at position 20 but were allowed to start at any position along the SL sequence. This approach captured possible alternative variants of the SL sequence present in the more distantly related *P. ovalis* genome. The genomic sequence of partial SL hits in *P. ovalis* were extracted using SeqKit (v0.15.0; Shen et al. 2016) along with enough of the bases upstream so that the final sequence retrieved was 20 bp in length (i.e., the length of the SL sequence in KR01). The extracted sequences that were <20 bp in length were discarded (e.g., hits near scaffold ends), and iqtree (v1.6.12; ‐m JC; Nguyen et al. 2015) was used to construct a maximum‐likelihood tree that was visualized, along with the extracted sequences, by iTOL (Letunic and Bork 2021) to determine if variants of the SL sequence were present in this species.

## Results and Discussion

### Survey of trans‐SL sequences in *Paulinella* using aligned RNA‐seq data

RNA‐seq reads (hereinafter, the standard RNA‐seq dataset; generated by Lhee et al. 2021 that aligned to KR01 gene CDSs and encoded the shorter SL sequence [CCGGCTTTTCTG]; see Materials and Methods) were used to identify the position along the CDS (the SL addition site) where the SL sequence was added. Using this approach, 8,423 SL addition sites were identified in 6,579 genes (20.33% of the 32,361 predicted genes in this species); 4,522 of the SL addition sites were located at position 1 of the CDS, meaning that the SL sequence was added either before the first base of the CDS or within one read length of the start of the CDS (i.e., the 5′ UTR; see Materials and Methods for more information). Each of these SL addition sites was supported by >10 mapped reads that contained the conserved SL region; there were no putative mispredicted regions within 100 bp of the SL addition sites. These sites are supported by an average of 35.47% of the aligned reads at that position and occur on average 10.55% of the length from the 5′‐ to the 3′‐end of transcripts (Fig. 1A). SL addition sites with above average (>35.47%) read support were positioned 2.53% of the length of the CDS, whereas sites with below average (<35.47%) support were positioned, on average, 19.49% of the way along the CDS (Fig. 1, B and C). We found 406 genes with SL addition sites (6.17% of 6,579 total SL addition site containing genes) that were downstream or overlapped annotated functional domains and were not interrupted by mispredicted regions. A total of 631 (9.59%) genes had SL addition sites that were downstream or overlapped annotated functional domains, transmembrane domains, or regions predicted as forming a coil structure (i.e., presumably functional). There was a significant difference (two–tailed *t*‐test, *t*_7157_ = −7.81, *p*‐value = 6.35 × 10^−15^) in the mean TPM levels of the genes with and without SL addition sites. The mean expression level of genes with SL addition sites was higher (62.95) than genes without SLs (22.72).

Of the 3,901 SL addition sites not positioned at the start (1^st^ position) of a KR01 CDS, 2,237 (57.34%) were at an exon junction. In the majority (90.9% of 2,237 and 52.14% of 3,901 total) of these sites, the SL sequence was added precisely at the exon splice site: i.e., the end of the SL sequence is followed by the first base of the exon to which the SL is attached. Of the 1,664 SL addition sites that did not overlap with exon boundaries (42.66% of 3,901 total), 1,358 did not occur close to the end of the transcript and could therefore be used to construct a sequence logo. These logos predictably showed a preference for addition at sites with the canonical splice site acceptor pattern AG (Fig. 2). Our results demonstrate that SLTS has an affinity for the same splice sites as the canonical cis*–*splicing mechanism and can utilize existing exon splice sites for SL addition. This suggests that SLTS may compete with *cis* splicing for access to donor–first acceptor sites, as previously reported (Hastings 2005, Zhang et al. 2007).

In *Paulinella* *chromatophora*, 4,006 SL addition sites (with support from >10 mapped reads) were found in 3,921 CDSs (6.47% of 60,559 predicted genes); 63 SL addition sites in 59 CDSs (0.097%) were positioned >100 bp from the 5′‐end of each sequence. There is a significant difference in the amount of RNA‐seq data available from these two *Paulinella* species. A single RNA‐seq library comprising 8,557,030 trimmed and filtered read pairs exist for *P. chromatophora*, whereas for KR01, there are 42 libraries with 620,702,822 such read‐pairs.

### “Noisy” trans‐splicing in *Paulinella*

Given the complex KR01 gene structures (Lhee et al. 2021), it is possible that SL addition distal from the 5′‐end of transcripts, in particular, at donor–first acceptor sites, is due to transcriptional noise. Up to 92–98% of observed splice variants (alternatively spliced transcripts) are generated by errors in the splicing process (Saudemont et al. 2017). This “noisy splicing” model posits that most transcripts generated by alternative splicing are non‐functional, with neutral or slightly deleterious fitness effects. If we assume a similar “noisy trans‐splicing” model in *Paulinella*, then most, but not all, of the conserved SL addition sites produce non‐functional transcripts that have minimal evolutionary impact. We find that in KR01, SL addition sites with higher‐than‐average read support are positioned closer to the (canonical) 5′‐end of transcripts than sites with lower than average read support. The latter may represent transcriptional noise because they are located further away from the 5′‐end of CDSs and are more likely to disrupt functional domains: observed in 9.59% of SL–containing transcripts.

The “noisy splicing” model also predicts that splicing rate error will be lower for highly expressed genes to prevent loss of resources on non‐functional transcripts. Although our analysis finds that transcripts of genes with SL addition sites had higher expression levels, we would need to identify all SL addition sites along each gene, including those in the UTR, before we can assess if genes with a higher expression level have fewer SL–truncated variants. We are also more likely to identify SL addition sites in genes with higher expression levels, which might explain why SL–containing genes have higher expression levels in KR01, and why we identified more SL addition sites in KR01 (42 RNA‐seq libraries) than *Paulinella* *chromatophora* (one library). The consequences of this competition for gene expression remain to be explored. Many of the SL addition sites we found may reflect transcriptional noise. This does not however invalidate our hypothesis that SLTS is capable of generating alternative transcripts, giving rise to novel proteins that can be acted upon by natural selection.

### Role of SLTS in plastid endosymbiosis

In KR01, 50 EGT–derived genes were identified by Lhee et al. (2021), 17 of which have SL addition sites (>10 mapped reads) that were detected by screening the standard RNA‐seq dataset. There are 32 EGT–derived genes that are annotated as encoding high light–inducible (Singer et al. 2017) proteins, of which only three have SL addition sites. Of the remaining 18 non‐HLI encoding genes, 14 (77.8%) have SL addition sites (Table S1 in the Supporting Information). One EGT–derived gene lacking an identified SL addition site is located at the start of a scaffold and may be truncated as a result. Four genes have SL addition sites near mispredicted regions. It is possibly that these represent instances where the 5′‐end of the CDS has been incorrectly predicted to extend into, and in some cases beyond, the 5′ UTR; in these cases, the SL addition sites may mark the correct 5′‐end of the transcript. Finally, six genes have multiple SL addition sites. Moreover, 53/98 (54.1%) of HGT–derived genes in KR01 have SL addition sites (Table S1), 16 have multiple SL addition sites and 24 are near mispredicted regions. Some genes are close to scaffold ends and may be truncated, preventing the identification of additional SL addition sites containing genes. The number of EGT or HGT genes with SL addition sites will also likely increase when gene models with UTRs become available. These results suggest that SLTS in *Paulinella* may have facilitated the activation and fixation of foreign genes in the host genome. To test this hypothesis, we searched for SL sequences in *Paulinella* genome assemblies to determine if SL RNA genes, required for SLTS, are present in each species. There were 151 hits that cover the full SL sequence in KR01, 52 hits in *P. chromatophora*, and none in the single‐cell draft genome assemblies (Bhattacharya et al. 2012) of *P*. *ovalis*, the heterotrophic sister lineage of the photosynthetic *Paulinella* species. In the latter, 118 hits partially cover the SL sequence but averaged only 10.7 bp in length and no conserved motifs were found in the upstream variable regions (Fig. S3 in the Supporting Information). The high copy number of SL RNA genes in photosynthetic *Paulinella* suggests that despite the *P. ovalis* genome being highly fragmented, we would expect (if present) to find evidence of their existence in the heterotrophic sister lineage. These observations suggest that the SL RNA genes in photosynthetic *Paulinella* are absent in *P. ovalis*. Therefore, *P*. *ovalis* either does not perform SLTS or uses SL RNA genes with divergent sequences. The former would suggest that the SLTS mechanism evolved *de novo* in photosynthetic *Paulinella*, whereas the latter might be a result of the fragmented and incomplete nature of the *P. ovalis* single‐cell genome assemblies and the lack of a comprehensive RNA‐seq dataset from which novel SL RNA genes can be characterized.

The biological function of splice leader sequences in *Paulinella* is unknown (Nowack et al. 2016, Matsuo et al. 2018). They could enhance expression, promote translation, or prevent the degradation of transcripts that contain these sequences. During the early stages of plastid endosymbiosis in *Paulinella*, there was an initial period of rapid gene loss and transfer from the chromatophore (Lhee et al. 2019). If SLTS had evolved in the ancestor of photosynthetic *Paulinella*, then it might have enhanced the rate at which EGT genes were retained, thereby facilitating integration and transfer of control of critical endosymbiont systems to the host. The EGT–derived HLI genes in KR01 have undergone gene family expansion and are encoded in outward–facing pairs (Lhee et al. 2021). The unique configuration and evolutionary history of this gene family suggest their integration into host biology, no longer requiring SLs for efficient expression. We hypothesize that due to the presence of this mechanism only in photosynthetic *Paulinella* and the high rate of SL addition on EGT– and HGT–derived gene transcripts, that SLTS has played a significant role in the evolution of plastid endosymbiosis in this lineage and that it might continue to play a role in the metabolic integration of the nascent organelle.

### Functional annotation of SL–containing transcripts in KR01

To gain additional insights into trans*‐*splicing in KR01, we sequenced two additional cDNA libraries using a cap–switching protocol that captures the 5′‐end of transcripts (Fig. 1D). This method generated 90,697 total transcripts, of which 669 contain a SL sequence. Of the 669 SL–containing sequences, 357 are unique to the cap–switching assembly (i.e., absent in the standard RNA‐seq dataset; Lhee et al. 2021). Of the 4,419 total SL–containing transcripts (357 unique to the cap–switching data and 4,062 generated from the standard RNA‐seq data; Lhee et al. 2021) 3,854 and 565 mapped to an annotated or unannotated locus in the genome, respectively (Table S2 in the Supporting Information). SL transcripts mapped to unannotated loci likely represent gene models absent from the available data, demonstrating how analysis of SL–containing transcripts can improve gene prediction, as shown previously for dinoflagellates (Zhanga et al. 2013). A total of 1,033 (26.8% of 3,854) trans*‐*spliced transcripts had blast hits to proteins with known functions (Table S2). The majority (2,822/3,854) of genes that overlap with mapped SL–containing transcripts are functionally annotated as either hypothetical, “predicted genes” (with unknown function), or have no blast hits. The presence of SLs on transcripts derived from dark genes and genes with organelle functions validates the existence of the former, and more generally, the ubiquity of this process in *Paulinella*.

**Fig. 3:**
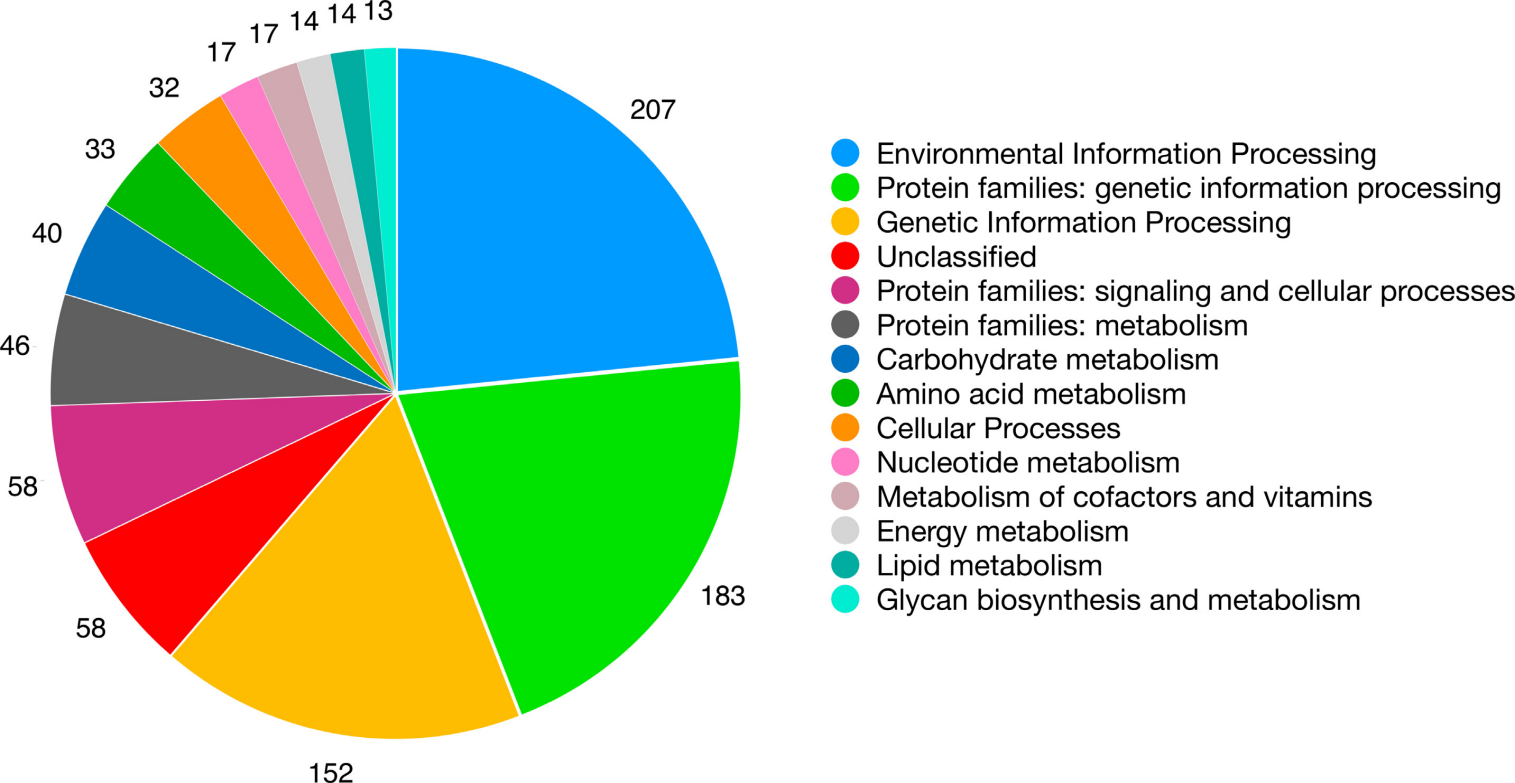
Classification of putative functions encoded by SL trans‐spliced transcripts. The putative functions are derived from KEGG functional categories; only categories with >10 proteins are shown. [Color figure can be viewed at wileyonlinelibrary.com]

KEGG categorical functions were assigned to 890 SL–containing transcripts (Fig. 3), covering a wide range of metabolic functions, with the most prominent being “environmental information processing” (which include genes involved in “environment adaptation” and “signaling transduction”) and “membrane transport”; the category “genetic information processing” includes proteins involved in transcription, translation, and DNA replication and repair. As previously found (Bitar et al. 2013), functional bias did not exist in the cellular function of genes that contain SL sequences. Analysis of SL–containing transcripts (compared to all assembled transcripts, independently of predicted reference genes) showed enrichment of Biological Process GO terms related to cell morphology and membrane transport, however, the relatively high (unadjusted) *p*‐value of these terms (>1 × 10^−6^) and the fragmented nature of the assembled transcripts makes it difficult to discern the biological significance of these results (Table S3 in the Supporting Information).

### SLTS is involved in alternative splicing of mRNAs generating SL–truncated transcripts

**Fig. 4:**
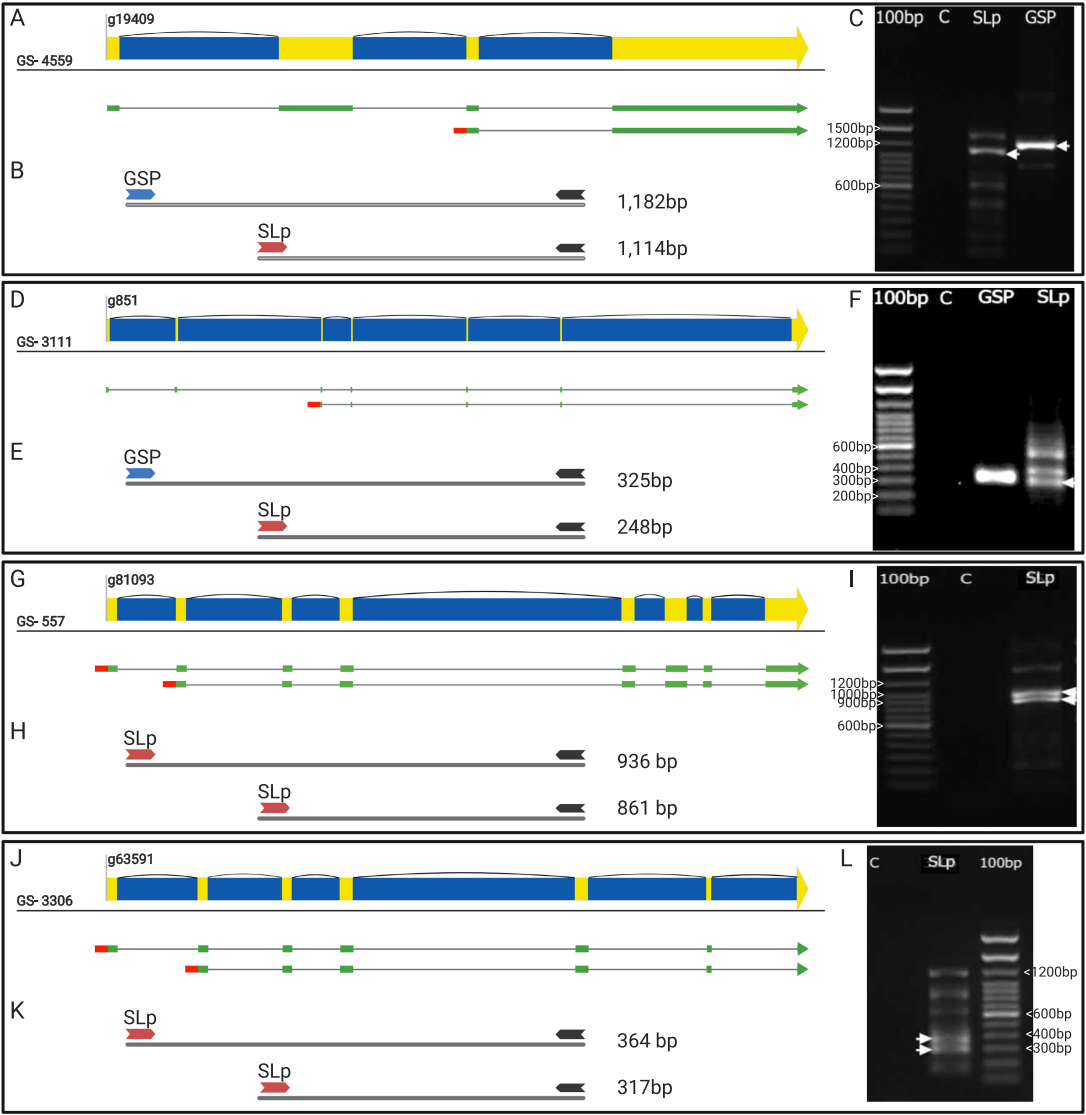
Examples of alternatively spliced transcripts in KR01. Panels A, D, G, J: four different gene profiles in different genome scaffold (GS). Transcript variants (green bars) are mapped under the corresponding annotated gene in the KR01 genome. The alternative truncated transcript variants (marked with red 5 ′‐ends ) caused by SL addition are also shown below the full‐length versions in each panel. Panels B, E, H, K: the expected sizes of PCR products using spliced leader–specific primer (SLp) or gene–specific primers (GSP) are shown. The associated gel image (C, F, I, L) shows the RT‐PCR products along with a negative control (C) lane. The white arrowheads indicate the target product as confirmed by DNA sequencing. This figure was partially created with BioRender.com; original uncropped gel images are shown in Figures S1 and S2. [Color figure can be viewed at wileyonlinelibrary.com]

Of the 3,854 SL–containing de novo transcripts that map to annotated loci, 3,626 encode the conserved region of the SL sequence with no mismatches ≤15 bp from the 5′‐end of the transcript (Fig. 1D). PASA aligned and validated 3,255 transcripts; 2,090 of which have the same intron structure as a gene from the reference genome. Transcripts were aligned against the associated reference gene CDS using BLASTn. Only transcripts with an alignment that started >100 bp along the reference gene CDS that started ≤2 bp along the transcript and that ended either >95% along the transcript or >95% along the CDS were retained. This produced a final set of 432 SL–containing transcripts that truncate (start part way along) a predicted reference gene. The bulk of SL–containing transcripts that failed at this stage did so because they do not truncate a reference gene (i.e., the 5′‐end of the SL–containing transcript is close [≤100 bp] to or upstream of the start of the reference gene). Of the 432 putative examples of genes with SL–truncated transcripts, 57 had consistent read mapping both up and downstream of the SL addition site (i.e., the region around the SL addition site is free from mispredictions); 43 remained after manual inspection (Fig. 1D; Table S4 in the Supporting Information). SL–truncated transcripts can be categorized into two types. Type 1 comprises SL transcripts in which the majority of reads at an SL truncation point are from the SL encoding transcript (i.e., the shorter SL truncated transcript variant has a higher expression than the long transcript variant). Type 2 comprises SL transcripts in which the minority of the reads at an SL truncation point are from the SL encoding transcript (i.e., the shorter SL truncated transcript variant has a lower expression than the long transcript variant). Of the 43 manually verified genes, 20 are Type 1 and 23 are Type 2. The presence of SL–truncated transcript variants was confirmed for four genes using RT‐PCR and sequencing (Figs. 4, S1 and S2); two of these genes are part of the 43 manually inspected genes (Table S4). Figure 4 (panels G and J) shows that two of the selected genes contain evidence for SL addition both in the long and truncated transcript variants. These transcripts were amplified using SL‐primers to generate both predicted PCR products (Fig. 4, I and L). The other two genes showed that only the truncated transcripts contains an SL sequence (Fig. 4, A and D). In these cases, gene–specific primers (GSP) were made for each transcript and used with the SL‐primers to generate PCR products (Fig. 4, B and E) that were sequenced to confirm the transcript variants.

### SL addition may generate novel functional proteins

**Fig. 5:**
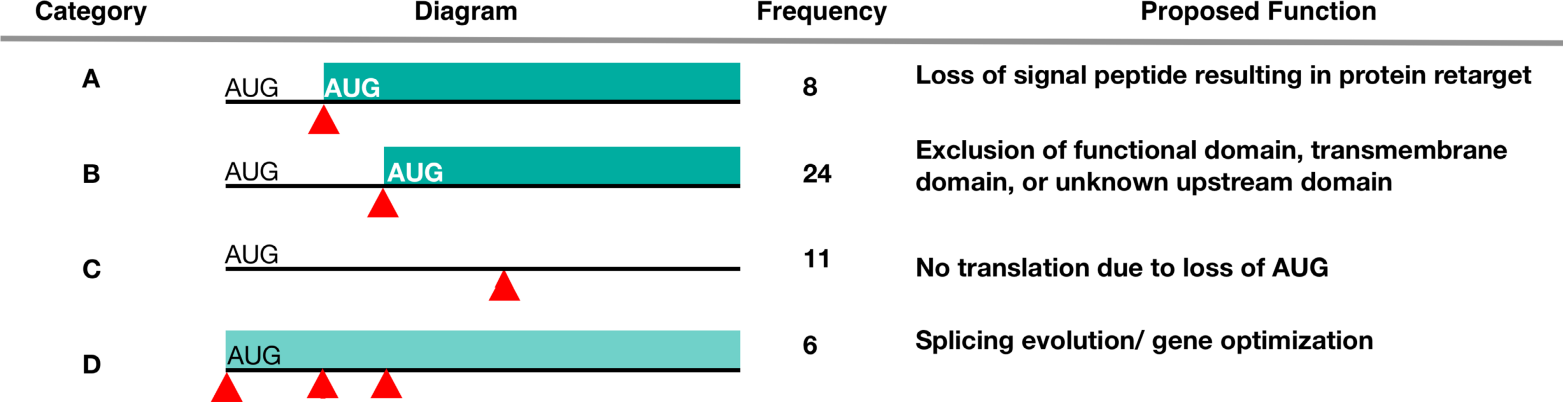
Proposed functions for SL trans‐spliced alternative transcripts. Alternative splice sites are indicated with the red arrowheads on the corresponding mRNA (black line) that contains an alternative open reading frame (bar). [Color figure can be viewed at wileyonlinelibrary.com]

To assess the functional consequences of SL addition at sites that interrupt the coding sequence of predicted genes, we examined the 43 manually curated genes that give rise to transcripts truncated by an SL. None of these candidates show evidence of misprediction around the SL addition site. Based on the differences in predicted functions of the SL–truncated transcripts compared to the full‐length versions, they fall into four categories. Initially, we investigated if the spliced variants were translatable (i.e., contained a downstream ORF). About eleven genes had SLTS transcripts that may result in translation suppression (Category C; Fig. 5). Eight had transcripts that lack an in‐frame start codon (AUG) downstream of the SL addition site, and three had transcripts that are significantly shorter than the full‐length variant. The latter set may or may not be functional even if an in‐frame start codon was present. The remaining proteins are translatable (i.e., contain an in‐frame start codon), despite lacking ~1–3 upstream exons. To better understand the consequences of SL addition in this group, we annotated the predicted proteins with functional domains, transmembrane helixes, and organelle transport signals. Four of the proteins were predicted by MITOPROT II and verified by iPSORT and DeepLoc‐1.0 to be targeted to the mitochondria. In these three cases, the addition of the internal SL sequence eliminated the targeting peptide and rendered the protein cytoplasmic (Category A; Fig. 5). This was also the case for one crTP, in which the *Paulinella*–specific leader sequence (which is ~200 aa in length; Singer et al. 2017) was eliminated by SL addition at the 5th exon. One protein had its localization changed from the cytoplasm to the extracellular space and three proteins had an InterProt predicted signal peptide, with an unclear transport destination, removed by SL addition (Table S4). This suggests that SLTS in *Paulinella* could cause retargeting of proteins. The majority of proteins are in Category B, whereby the addition of an SL sequence eliminates an upstream domain which could impact protein function (e.g., binding specificity; Uddin et al. 2016), structure (i.e., membrane bound or soluble; Alberts et al. 2002), localization, post‐translational modification, and/or regulation (Arnesen 2011, Varland et al. 2015). Category D transcripts provide evidence of SL addition at multiple sites and may include transcripts classified under Categories A‐C. Although it is unclear why some genes have multiple SL addition sites, it is possible that this process is driven by selection for novel transcript variants. It is also possible that the SLTS is less specific than cis*‐*splicing and may add SL sequences to multiple off‐target locations such as those located in close proximity.

### A putative model for SL driven gene origination

**Fig. 6:**
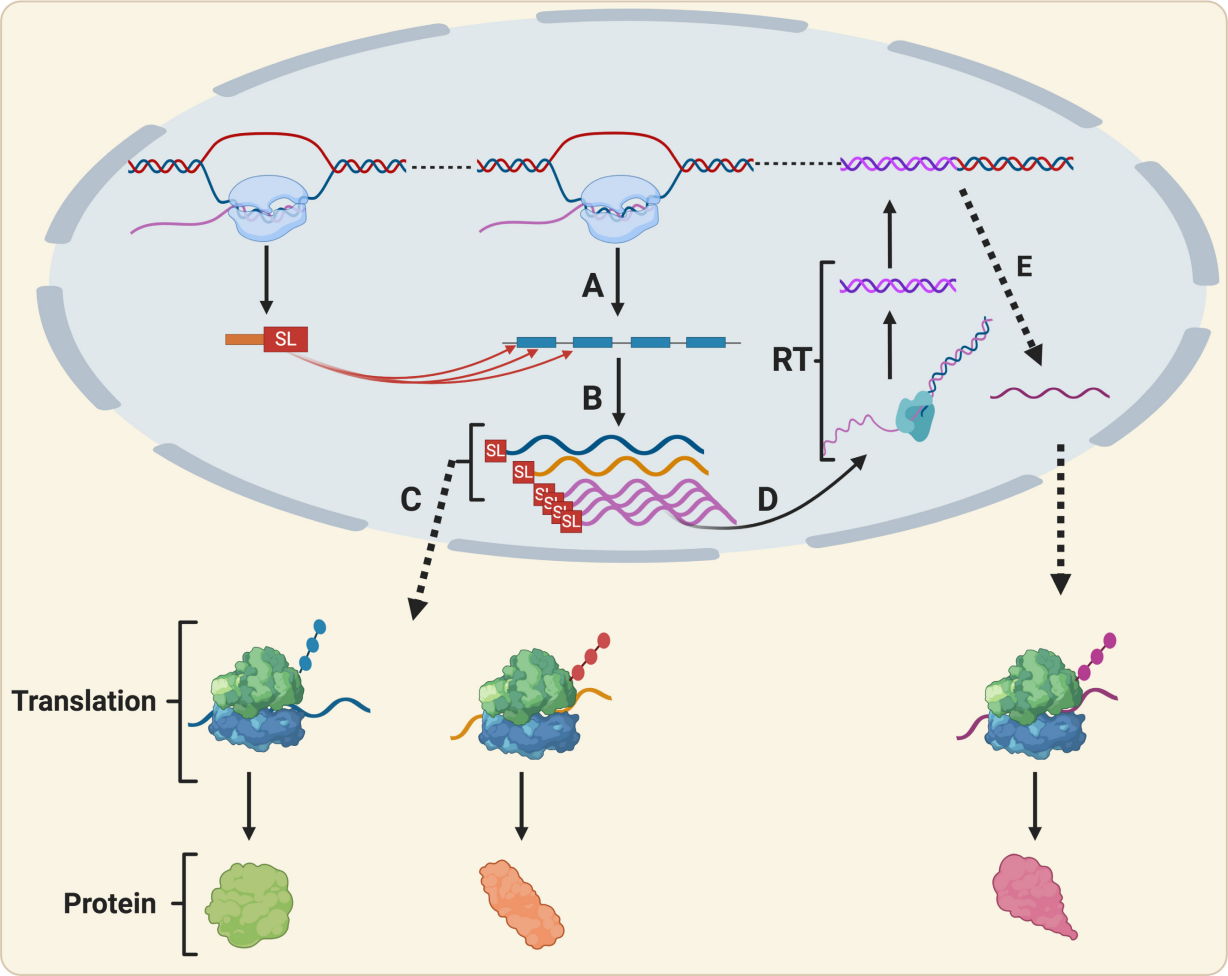
Proposed model for SL addition as a mechanism for novel gene origin. (A) SL donated from non‐coding (nc)RNA is added into different locations on a pre‐mRNA as depicted by the red arrows. (B) Alternative spliced variants are generated resulting in changes (C) in gene product. (D) Highly expressed variants undergo mRNA recycling back into the genome via reverse transcription (RT) and non‐homologous recombination, leading to the possible fixation and (E) generation of a novel gene. Created with BioRender.com. [Color figure can be viewed at wileyonlinelibrary.com]

If the likelihood of SL addition within the coding region of a transcript is dependent on gene expression level, higher expression would result in more addition sites, and more novel variants. This would putatively drive functional innovation in highly expressed genes. In dinoflagellates, reverse transcription and non‐homologous recombination drive the recycling of mRNA that contains SL sequences (Slamovits and Keeling 2008, Stephens et al. 2020). In this process, an mRNA is reverse transcribed to cDNA and is integrated into the genome through recombination, resulting in an intron–lacking duplicate locus that, if transcribed, may yield mRNA with one or multiple additional SL sequences (Slamovits and Keeling 2008, Jaeckisch et al. 2011). In KR01, there were 11 candidate genes that showed evidence of mRNA recycling. Of these, six passed manual verification, and two were single‐exon genes (Table S5 in the Supporting Information). This suggests that mRNA recycling likely occurs in *Paulinella* and can act as a mechanism for fixing novel (truncated) transcript variants in the genome. We hypothesize that these two mechanisms (SL truncation and mRNA recycling) provide pathways for generating and fixing novel variants of highly expressed or adaptive genes in the *Paulinella* genome (Fig. 6). Furthermore, this mechanism may allow genes to acquire new promoters with different expression patterns, or in the case of genes acquired via EGT or HGT, to gain more efficient eukaryotic promoters (Casola and Betran 2017). It is also possible that the SL sequence acts as a promoter for the genes integrated into the genome, similar to what has been proposed in dinoflagellates (Song et al. 2017); however, this hypothesis remains to be studied in more detail once the promoter regions in *Paulinella* are characterized. In comparison to processes such as gene duplication which is impacted by the genomic context, such as proximity to transposable elements, SL truncation could act as a direct method for generating novel, potentially adaptive, genes and associated functions.

### Challenges faced when studying SL addition

The number of SL addition sites identified in KR01 is likely an underestimate of their true frequency. SLTS normally occurs upstream of the coding region; one of our approaches for identifying SL addition sites, which uses short reads aligned against the CDS of the predicted protein–coding genes (that lack annotated UTR regions), is limited to detecting SL sequences that are added to transcripts within the coding region or immediately (less than the length of a full read) upstream of the predicted start codon. Our other approach, which involved assembling the RNA‐seq data de novo before extracting transcripts with SL sequences, would be able to identify SL addition sites in the UTRs of genes. However, this approach identified fewer genes with SL addition sites than the read–based approach, likely because transcript assembly programs are not designed to handle SL–containing transcripts, so it cannot be assumed that they will recover all variants present in the data, particularly, if they are in relatively low abundance.

In a number of genes with SL–truncated transcripts, we observe regions (particularly at the 5′‐end) that show evidence of misprediction. This is likely a result of the challenges faced when predicting the correct start codon using an ab initio approach (as done for the KR01 reference genome). In many cases, the N‐terminus of the predicted reference proteins was erroneously extended into and beyond the 5′‐UTR region (resulting in poor read mapping). The use of short‐read RNA‐seq data can also result in ambiguous placement of SL addition sites caused by reads that align to homologous gene regions. In our analysis of genes with SL–truncated transcripts, we verified that the region around the SL addition site of interest was not mispredicted and that both reads in a read pair were aligned in the correct orientation, reducing the chances of detecting false or ambiguous SL addition sites within coding regions. These issues make it more challenging to discern the functional consequences of SL–based transcript truncation. We are nonetheless confident in our results because the observed deviations from the expected positioning of SL addition sites along transcripts provide strong evidence that multiple transcripts are derived from the same gene as a result of SLTS, irrespective of whether SL addition occurs within the coding region or UTR. The only way to identify the true start codon for each full‐length and SL–truncated protein is through proteomics or through advanced translation start site capture techniques that have not yet been established in our model. There is also a need for deep sequencing of expressed transcripts using long‐read–based technologies (e.g., PacBio IsoSeq) which produce reads that can be unambiguously aligned against the available gene models, thereby accurately revealing the structure and location of SL addition sites along a transcript. Our analysis focuses on the potential effects of SL addition on resulting proteins. We did not assess the potential effects that removal of the 5′ untranslated region from a transcript may have on translational efficiency or stability.

## Conclusions

Our work suggests that the addition of SL sequences at sites within the coding region of KR01 transcripts occurs frequently and may result from “noisy trans‐splicing.” SLTS is implicated in the integration of novel “dark” genes and genes of foreign origin acquired via EGT or HGT. These results lead us to hypothesize that trans*‐*splicing of nuclear–derived transcripts in *Paulinella* can generate novel or altered proteins that can be acted upon by natural selection, facilitating the integration of the novel photosynthetic organelle, the chromatophore.

##  

AG, TGS, and DB were supported by a grant from the National Aeronautics and Space Administration (80NSSC19K0462) awarded to DB. DB was supported by a NIFA‐USDA Hatch grant (NJ01180). We declare no conflicts of interest.

## Author Contributions

**A. Gabr:** Conceptualization (equal); Formal analysis (equal); Investigation (equal); Methodology (equal); Writing – original draft (equal); Writing – review & editing (equal). **T.G Stephens:** Conceptualization (equal); Formal analysis (equal); Investigation (equal); Methodology (equal); Writing – original draft (equal); Writing – review & editing (equal). **D. Bhattacharya:** Conceptualization (equal); Funding acquisition (equal); Project administration (equal); Supervision (equal); Writing – original draft (equal); Writing – review & editing (equal).

## Supporting information


**Figure S1**. Examples of alternatively spliced transcripts in the KR01 genome.

**Figure S2**. Examples of alternatively spliced transcripts in the KR01 genome.

**Figure S3**. A maximum likelihood phylogenetic tree constructed using sequences extracted from partial SL hits to the *Paulinella  ovalis* genome (visualization by iTOL).

**Table S1**. EGT and HGT derived genes in KR01 with info about SL addition sites shown when present.

**Table S2**. Annotation of genes that contained SL transcripts.

**Table S3**. GO terms enriched in SL‐containing transcripts compared against all transcripts that aligned to the KR01 genome.

**Table S4**. List of genes that passed the stringent filtering criteria and were subject to manually inspection.

**Table S5**. Candidate genes for mRNA recycling.

## Data Availability

The RNA‐seq data from KR01 are available from the NCBI’s SRA repository (BioProject ID PRJNA568118). RNA‐seq data generated from 5′RACE are available from the NCBI’s SRA repository (BioProject ID PRJNA730897). Assembled standard and cap–switching transcripts are available from https://doi.org/10.5281/zenodo.5703668. All other data supporting the conclusions of this article are included within the article (and its additional file). Code to reproduce our analyses can be found here: https://github.com/TimothyStephens/Paulinella_spliced_leader_sequence
