# Supplementary material for: Hypothesis: Trans‐splicing Generates Evolutionary Novelty in the Photosynthetic Amoeba Paulinella
Source: J Phycol. 2022 Mar 25;58(3):392–405. doi: 10.1111/jpy.13247 (PMC9311404; doi:10.1111/jpy.13247)
Supplement: Supplementary file 2 — Figure S2. Examples of alternatively spliced transcripts in the KR01 genome. [file JPY-58-392-s002.pdf]

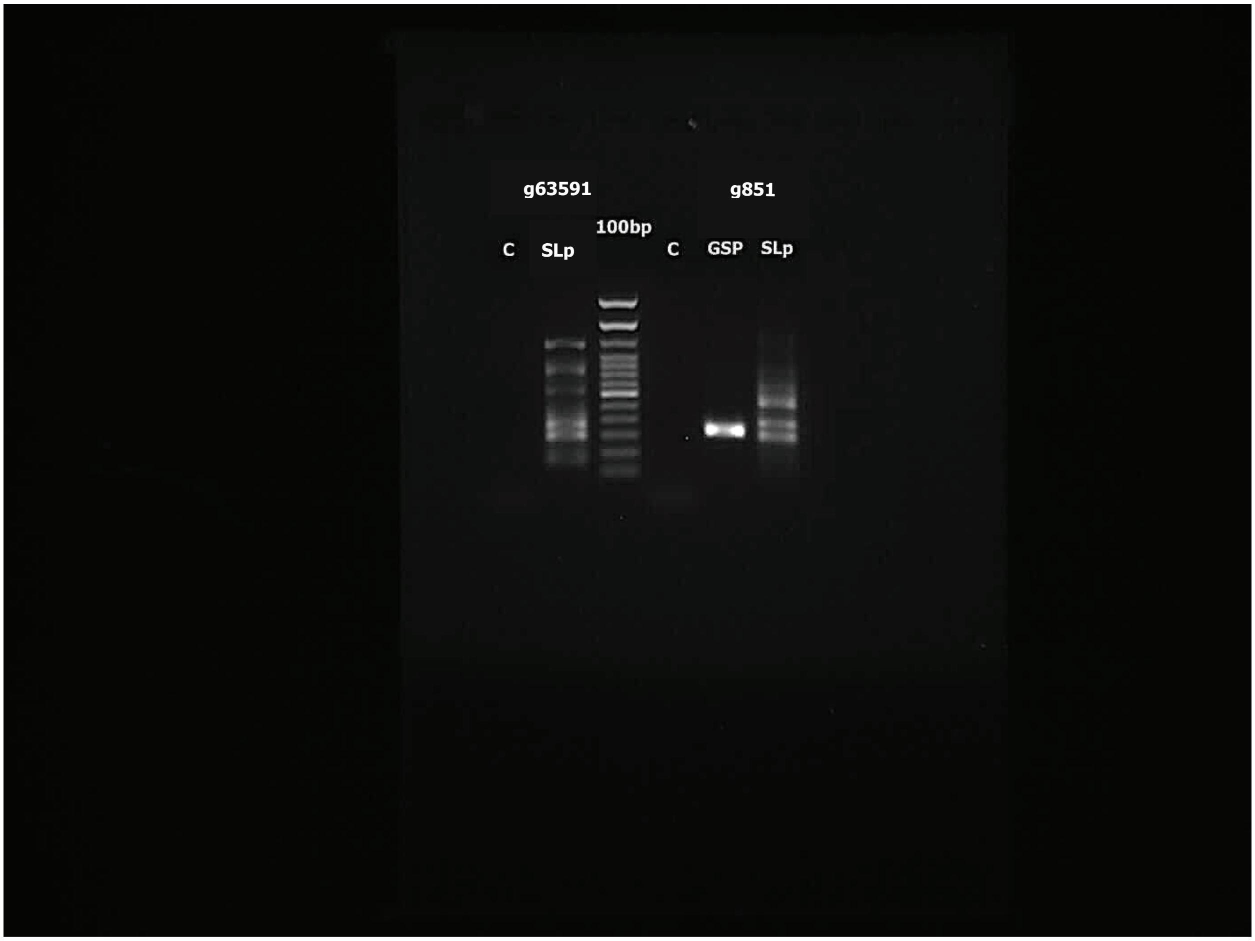

Supplementary Figure S2. Examples of alternatively spliced transcripts in the KR01 genome. Full length image of Figure 4 associated gel (L , F) shows the RT-PCR products.
