# Supplementary material for: Hypothesis: Trans‐splicing Generates Evolutionary Novelty in the Photosynthetic Amoeba Paulinella
Source: J Phycol. 2022 Mar 25;58(3):392–405. doi: 10.1111/jpy.13247 (PMC9311404; doi:10.1111/jpy.13247)
Supplement: Supplementary file 3 — Figure S3. A maximum likelihood phylogenetic tree constructed using sequences extracted from partial SL hits to the Paulinella ovalis genome (visualization by iTOL). [file JPY-58-392-s007.pdf]

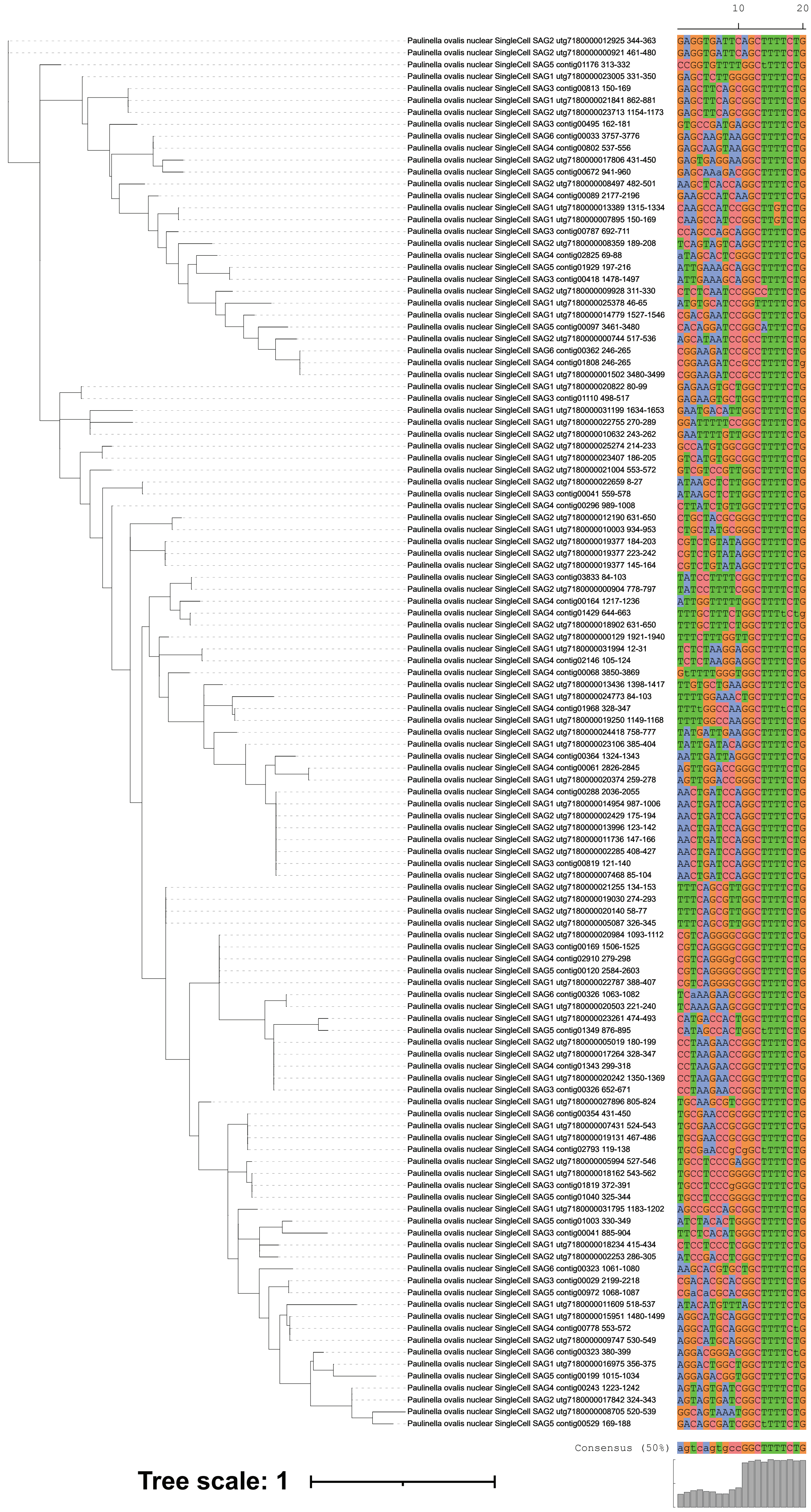

10
20
Paulinella ovalis nuclear SingleCell SAG2 utg7180000012925 344-363 GAGGTGATTCAGCTTTTCTG
Paulinella ovalis nuclear SingleCell SAG2 utg7180000000921 461-480 GAGGTGATTCAGCTTTTCTG
Paulinella ovalis nuclear SingleCell SAG5 contig01176 313-332 CCGGTGTTTTGGCtTTTCTG
Paulinella ovalis nuclear SingleCell SAG1 utg7180000023005 331-350 GAGCTCTTGGGGCTTTTCTG
Paulinella ovalis nuclear SingleCell SAG3 contig00813 150-169 GAGCTTCAGCGGCTTTTCTG
Paulinella ovalis nuclear SingleCell SAG1 utg7180000021841 862-881 GAGCTTCAGCGGCTTTTCTG
Paulinella ovalis nuclear SingleCell SAG2 utg7180000023713 1154-1173 GAGCTTCAGCGGCTTTTCTG
Paulinella ovalis nuclear SingleCell SAG3 contig00495 162-181 GTGCCGATGAGGCTTTTCTG
Paulinella ovalis nuclear SingleCell SAG6 contig00033 3757-3776 GAGCAAGTAAGGCTTTTCTG
Paulinella ovalis nuclear SingleCell SAG4 contig00802 537-556 GAGCAAGTAAGGCTTTTCTG
Paulinella ovalis nuclear SingleCell SAG2 utg7180000017806 431-450 GAGTGAGGAAGGCTTTTCTG
Paulinella ovalis nuclear SingleCell SAG5 contig00672 941-960 GAGCAAaGACGGCTTTTCTG
Paulinella ovalis nuclear SingleCell SAG2 utg7180000008497 482-501 AAGCTCACCAGGCTTTTCTG
Paulinella ovalis nuclear SingleCell SAG4 contig00089 2177-2196 GAAGCCATCAAGCTTTTCTG
Paulinella ovalis nuclear SingleCell SAG1 utg7180000013389 1315-1334 CAAGCCATCCGGCTTGTCTG
Paulinella ovalis nuclear SingleCell SAG1 utg7180000007895 150-169 CAAGCCATCCGGCTTGTCTG
Paulinella ovalis nuclear SingleCell SAG3 contig00787 692-711 CCAGCCAGCAGGCTTTTCTG
Paulinella ovalis nuclear SingleCell SAG2 utg7180000008359 189-208 TCAGTAGTCAGGCTTTTCTG
Paulinella ovalis nuclear SingleCell SAG4 contig02825 69-88 aTAGCACTCGGGCTTTTCTG
Paulinella ovalis nuclear SingleCell SAG5 contig01929 197-216 ATTGAAAGCAGGCTTTTCTG
Paulinella ovalis nuclear SingleCell SAG3 contig00418 1478-1497 ATTGAAAGCAGGCTTTTCTG
Paulinella ovalis nuclear SingleCell SAG2 utg7180000009928 311-330 CTCTCAATCCGGCCTTTCTG
Paulinella ovalis nuclear SingleCell SAG1 utg7180000025378 46-65 ATGTGCATCCGGTTTTTCTG
Paulinella ovalis nuclear SingleCell SAG1 utg7180000014779 1527-1546 CGACGAATCCGGCTTTTCTG
Paulinella ovalis nuclear SingleCell SAG5 contig00097 3461-3480 CACAGGATCCGGCATTTCTG
Paulinella ovalis nuclear SingleCell SAG2 utg7180000000744 517-536 AGCATAATCCGCCTTTTCTG
Paulinella ovalis nuclear SingleCell SAG6 contig00362 246-265 CGGAAGATCCGCCTTTTCTG
Paulinella ovalis nuclear SingleCell SAG4 contig01808 246-265 CGGAAGATCcGCCTTTTCTg
Paulinella ovalis nuclear SingleCell SAG1 utg7180000001502 3480-3499 CGGAAGATCCGCCTTTTCTG
Paulinella ovalis nuclear SingleCell SAG1 utg7180000020822 80-99 GAGAAGTGCTGGCTTTTCTG
Paulinella ovalis nuclear SingleCell SAG3 contig01110 498-517 GAGAAGTGCTGGCTTTTCTG
Paulinella ovalis nuclear SingleCell SAG1 utg7180000031199 1634-1653 GAATGACATTGGCTTTTCTG
Paulinella ovalis nuclear SingleCell SAG1 utg7180000022755 270-289 GGATTTTTCCGGCTTTTCTG
Paulinella ovalis nuclear SingleCell SAG2 utg7180000010632 243-262 GAATTTTGTTGGCTTTTCTG
Paulinella ovalis nuclear SingleCell SAG2 utg7180000025274 214-233 GCCATGTGGCGGCTTTTCTG
Paulinella ovalis nuclear SingleCell SAG1 utg7180000023407 186-205 GTCATGTGGCGGCTTTTCTG
Paulinella ovalis nuclear SingleCell SAG2 utg7180000021004 553-572 GTCGTCCGTTGGCTTTTCTG
Paulinella ovalis nuclear SingleCell SAG2 utg7180000022659 8-27 ATAAGCTCTTGGCTTTTCTG
Paulinella ovalis nuclear SingleCell SAG3 contig00041 559-578 ATAAGCTCTTGGCTTTTCTG
Paulinella ovalis nuclear SingleCell SAG4 contig00296 989-1008 CTTATCTGTTGGCTTTTCTG
Paulinella ovalis nuclear SingleCell SAG2 utg7180000012190 631-650 CTGCTACGCGGGCTTTTCTG
Paulinella ovalis nuclear SingleCell SAG1 utg7180000010003 934-953 CTGCTATGCGGGCTTTTCTG
Paulinella ovalis nuclear SingleCell SAG2 utg7180000019377 184-203 CGTCTGTATAGGCTTTTCTG
Paulinella ovalis nuclear SingleCell SAG2 utg7180000019377 223-242 CGTCTGTATAGGCTTTTCTG
Paulinella ovalis nuclear SingleCell SAG2 utg7180000019377 145-164 CGTCTGTATAGGCTTTTCTG
Paulinella ovalis nuclear SingleCell SAG3 contig03833 84-103 TATCCTTTTCGGCTTTTCTG
Paulinella ovalis nuclear SingleCell SAG2 utg7180000000904 778-797 TATCCTTTTCGGCTTTTCTG
Paulinella ovalis nuclear SingleCell SAG4 contig00164 1217-1236 ATTGGTTTTTGGCTTTTCTG
Paulinella ovalis nuclear SingleCell SAG4 contig01429 644-663 TTTGCTTTCTGGCTTTtCtg
Paulinella ovalis nuclear SingleCell SAG2 utg7180000018902 631-650 TTTGCTTTCTGGCTTTTCTG
Paulinella ovalis nuclear SingleCell SAG2 utg7180000000129 1921-1940 TTTCTTTGGTTGCTTTTCTG
Paulinella ovalis nuclear SingleCell SAG1 utg7180000031994 12-31 TCTCTAAGGAGGCTTTTCTG
Paulinella ovalis nuclear SingleCell SAG4 contig02146 105-124 TCTCTAAGGAGGCTTTTCTG
Paulinella ovalis nuclear SingleCell SAG4 contig00068 3850-3869 GtTTTTGGGTGGCTTTTCTG
Paulinella ovalis nuclear SingleCell SAG2 utg7180000013436 1398-1417 TTGTGCTGAAGGCTTTTCTG
Paulinella ovalis nuclear SingleCell SAG1 utg7180000024773 84-103 TTTTGGAAACTGCTTTTCTG
Paulinella ovalis nuclear SingleCell SAG4 contig01968 328-347 TTTtGGCCAAGGCTTTtCTG
Paulinella ovalis nuclear SingleCell SAG1 utg7180000019250 1149-1168 TTTTGGCCAAGGCTTTTCTG
Paulinella ovalis nuclear SingleCell SAG2 utg7180000024418 758-777 TATGATTGAAGGCTTTTCTG
Paulinella ovalis nuclear SingleCell SAG1 utg7180000023106 385-404 TATTGATACAGGCTTTTCTG
Paulinella ovalis nuclear SingleCell SAG4 contig00364 1324-1343 AATTGATTAGGGCTTTTCTG
Paulinella ovalis nuclear SingleCell SAG4 contig00061 2826-2845 AGTTGGACCGGGCTTTTCTG
Paulinella ovalis nuclear SingleCell SAG1 utg7180000020374 259-278 AGTTGGACCGGGCTTTTCTG
Paulinella ovalis nuclear SingleCell SAG4 contig00288 2036-2055 AACTGATCCAGGCTTTTCTG
Paulinella ovalis nuclear SingleCell SAG1 utg7180000014954 987-1006 AACTGATCCAGGCTTTTCTG
Paulinella ovalis nuclear SingleCell SAG2 utg7180000002429 175-194 AACTGATCCAGGCTTTTCTG
Paulinella ovalis nuclear SingleCell SAG2 utg7180000013996 123-142 AACTGATCCAGGCTTTTCTG
Paulinella ovalis nuclear SingleCell SAG2 utg7180000011736 147-166 AACTGATCCAGGCTTTTCTG
Paulinella ovalis nuclear SingleCell SAG2 utg7180000002285 408-427 AACTGATCCAGGCTTTTCTG
Paulinella ovalis nuclear SingleCell SAG3 contig00819 121-140 AACTGATCCAGGCTTTTCTG
Paulinella ovalis nuclear SingleCell SAG2 utg7180000007468 85-104 AACTGATCCAGGCTTTTCTG
Paulinella ovalis nuclear SingleCell SAG2 utg7180000021255 134-153 TTTCAGCGTTGGCTTTTCTG
Paulinella ovalis nuclear SingleCell SAG2 utg7180000019030 274-293 TTTCAGCGTTGGCTTTTCTG
Paulinella ovalis nuclear SingleCell SAG2 utg7180000020140 58-77 TTTCAGCGTTGGCTTTTCTG
Paulinella ovalis nuclear SingleCell SAG2 utg7180000005087 326-345 TTTCAGCGTTGGCTTTTCTG
Paulinella ovalis nuclear SingleCell SAG2 utg7180000020984 1093-1112 CGTCAGGGGCGGCTTTTCTG
Paulinella ovalis nuclear SingleCell SAG3 contig00169 1506-1525 CGTCAGGGGCGGCTTTTCTG
Paulinella ovalis nuclear SingleCell SAG4 contig02910 279-298 CGTCAGGGgCGGCTTTTCTG
Paulinella ovalis nuclear SingleCell SAG5 contig00120 2584-2603 CGTCAGGGGCGGCTTTTCTG
Paulinella ovalis nuclear SingleCell SAG1 utg7180000022787 388-407 CGTCAGGGGCGGCTTTTCTG
Paulinella ovalis nuclear SingleCell SAG6 contig00326 1063-1082 TCaAAGAAGCGGCTTTTCTG
Paulinella ovalis nuclear SingleCell SAG1 utg7180000020503 221-240 TCAAAGAAGCGGCTTTTCTG
Paulinella ovalis nuclear SingleCell SAG1 utg7180000023261 474-493 CATGACCACTGGCTTTTCTG
Paulinella ovalis nuclear SingleCell SAG5 contig01349 876-895 CATAGCCACTGGCtTTTCTG
Paulinella ovalis nuclear SingleCell SAG2 utg7180000005019 180-199 CCTAAGAACCGGCTTTTCTG
Paulinella ovalis nuclear SingleCell SAG2 utg7180000017264 328-347 CCTAAGAACCGGCTTTTCTG
Paulinella ovalis nuclear SingleCell SAG4 contig01343 299-318 CCTAAGAACCGGCTTTTCTG
Paulinella ovalis nuclear SingleCell SAG1 utg7180000020242 1350-1369 CCTAAGAACCGGCTTTTCTG
Paulinella ovalis nuclear SingleCell SAG3 contig00326 652-671 CCTAAGAACCGGCTTTTCTG
Paulinella ovalis nuclear SingleCell SAG1 utg7180000027896 805-824 TGCAAGCGTCGGCTTTTCTG
Paulinella ovalis nuclear SingleCell SAG6 contig00354 431-450 TGCGAACCGCGGCTTTTCTG
Paulinella ovalis nuclear SingleCell SAG1 utg7180000007431 524-543 TGCGAACCGCGGCTTTTCTG
Paulinella ovalis nuclear SingleCell SAG1 utg7180000019131 467-486 TGCGAACCGCGGCTTTTCTG
Paulinella ovalis nuclear SingleCell SAG4 contig02793 119-138 TGCGaACCgCgGCtTTTCTG
Paulinella ovalis nuclear SingleCell SAG2 utg7180000005994 527-546 TGCCTCCCGAGGCTTTTCTG
Paulinella ovalis nuclear SingleCell SAG1 utg7180000018162 543-562 TGCCTCCCGGGGCTTTTCTG
Paulinella ovalis nuclear SingleCell SAG3 contig01819 372-391 TGCCTCCCgGGGCTTTTCTG
Paulinella ovalis nuclear SingleCell SAG5 contig01040 325-344 TGCCTCCCGGGGCTTTTCTG
Paulinella ovalis nuclear SingleCell SAG1 utg7180000031795 1183-1202 AGCCGCCAGCGGCTTTTCTG
Paulinella ovalis nuclear SingleCell SAG5 contig01003 330-349 ATCTACACTGGGCTTTTCTG
Paulinella ovalis nuclear SingleCell SAG3 contig00041 885-904 TTCTCACATGGGCTTTTCTG
Paulinella ovalis nuclear SingleCell SAG1 utg7180000018234 415-434 CTCCTCCCTCGGCTTTTCTG
Paulinella ovalis nuclear SingleCell SAG2 utg7180000002253 286-305 ATCCGACCTCGGCTTTTCTG
Paulinella ovalis nuclear SingleCell SAG6 contig00323 1061-1080 AAGCACGTGCTGCTTTTCTG
Paulinella ovalis nuclear SingleCell SAG3 contig00029 2199-2218 CGACACGCACGGCTTTTCTG
Paulinella ovalis nuclear SingleCell SAG5 contig00972 1068-1087 CGaCaCGCACGGCTTTTCTG
Paulinella ovalis nuclear SingleCell SAG1 utg7180000011609 518-537 ATACATGTTTAGCTTTTCTG
Paulinella ovalis nuclear SingleCell SAG1 utg7180000015951 1480-1499 AGGCATGCAGGGCTTTTCTG
Paulinella ovalis nuclear SingleCell SAG4 contig00778 553-572 AGGCATGCAGGGCTTTTCtG
Paulinella ovalis nuclear SingleCell SAG2 utg7180000009747 530-549 AGGCATGCAGGGCTTTTCTG
Paulinella ovalis nuclear SingleCell SAG6 contig00323 380-399 AGGACGGGACGGCTTTTCtG
Paulinella ovalis nuclear SingleCell SAG1 utg7180000016975 356-375 AGGACTGGCTGGCTTTTCTG
Paulinella ovalis nuclear SingleCell SAG5 contig00199 1015-1034 AGGAGACGGTGGCTTTTCTG
Paulinella ovalis nuclear SingleCell SAG4 contig00243 1223-1242 AGTAGTGATCGGCTTTTCTG
Paulinella ovalis nuclear SingleCell SAG2 utg7180000017842 324-343 AGTAGTGATCGGCTTTTCTG
Paulinella ovalis nuclear SingleCell SAG2 utg7180000008705 520-539 GGCAGTAAATGGCTTTTCTG
Paulinella ovalis nuclear SingleCell SAG5 contig00529 169-188 GACAGCGATCGGCtTTTCTG
Consensus (50%) agtcagtgccGGCTTTTCTG
Tree scale: 1

Supplementary Figure S3. A maximum likelihood phylogenetic tree constructed using sequences extracted from partial SL hits to the *P. ovalis* genome (visualization by iTOL). The sequences used to construct the tree are shown on the left of the tip labels; residues are colored using the ClustalX color scheme with a consensus sequence shown at the bottom of the alignment.
